# OPTICS-based Unsupervised Method for Flaking Degree Evaluation on the Murals in Mogao Grottoes

**DOI:** 10.1038/s41598-018-34317-7

**Published:** 2018-10-29

**Authors:** Pan Li, Meijun Sun, Zheng Wang, Bolong Chai

**Affiliations:** 1 School of Computer Science and Technology, Tianjin University, Tianjin, China; 2 School of Software, Tianjin University, Tianjin, China; 3 Dunhuang Academy, Gansu, China

## Abstract

In recent years, the preventive protection and restoration work of the murals in Mogao Grottoes has received extensive attention. Due to the fragility and detachment of the murals, it is necessary to study non-contact disease detection and prevention methods. In this paper, we propose an unsupervised method to accurately predict the degree of mural flaking diseases in Mogao Grottoes. The hyperspectral image (HSI) is captured by V10-PS hyperspectral camera. The proposed method includes three main steps: (1) extract the spectral features of the HSI by Principal Component Analysis (PCA) and Sparse Auto-Encoder (SAE) respectively; (2) cluster the extracted features by the Ordering Points to Identify the Clustering Structure (OPTICS) algorithm based on the density; (3) calculate the distance between the cluster core point and the other points in the feature space and visualize the final classification result. Different from other existing hyperspectral classification works, the research proposed in this paper is the degree detection of flaking of murals. Since the degree of flaking is continuous and the work is conducted without any supervision information, the entire workflow is complex and challenging. The experimental results show the effectiveness of our method.

## Introduction

HSI is generally obtained by visible light or near-infrared (NIR) hyperspectral camera. HSI not only contains the spatial information of the target object, but records the spectral domain information, which realize the organic combination of traditional imaging technology and spectrum detection technology and achieve the purpose of multi-dimensional information acquisition. Recently, HSI has been widely used for target detection^1–4^, agricultural monitoring^5–7^, environment surveillance^8–10^ and spectral unmixing^11,12^ based on spectral information. Due to the non-contact characteristics of HSI, hyperspectral imaging is gradually used in the field of cultural relic protection^13–15^ and has achieved good results. In the mid-1990s, HSI was first used to protect cultural heritage^16^, later, more and more countries and researchers began to use HSI for cultural relic detection and protection. Considering HSIs can capture abundant spectral information under non-contact conditions, which is of great significance for studying the state of cultural relics, we collected the murals of Mogao Grottoes using V10-PS hyperspectral camera with a spectral range of approximately 400 nm to 1000 nm.

**Figure 1:**
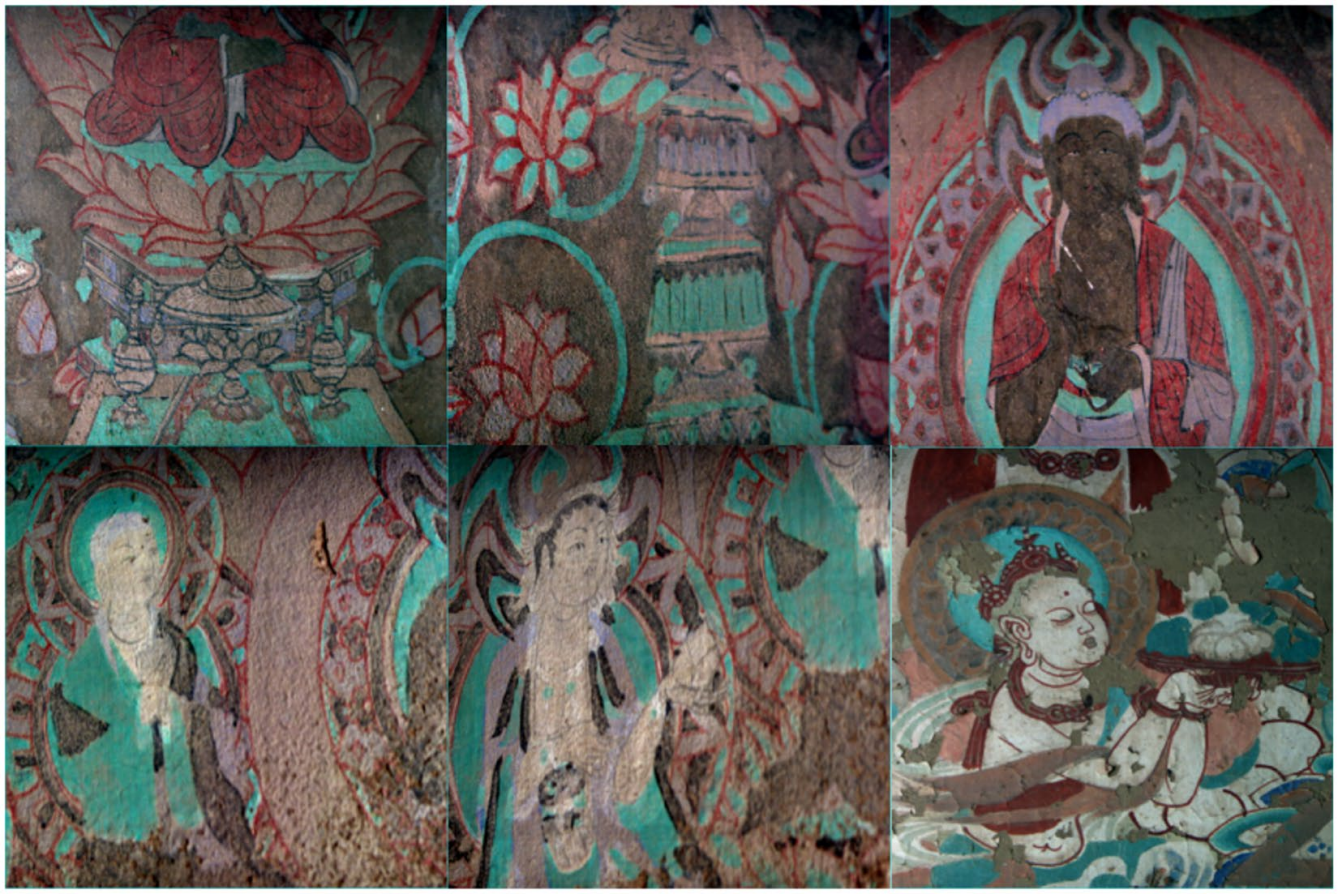
Some examples of murals at the Mogao Grottoes. Be grateful to Bolong Chai for taking these photos.

Mogao Grottoes murals, as an important historical and cultural heritage, have high archaeological, historical, literature, and religious research values. It is located in the westernmost part of Gansu Province, China with its history of more than 2000 years. Therefore, it is of great significance to use HSI to research and protect the murals of the Mogao Grottoes, especially to evaluate the flaking degree. Some examples of the murals of Mogao Grottoes are shown in Fig. 1.

Different from RGB image processing methods^17–19^, which aim to identify the main objects in the scene, HSI classification is a pixel2pixel task which purpose to assign a unique category label to each pixel in the HSI. In recent years, deep learning methods^20–22^, the support vector machines (SVMs)^23–25^, and semi-supervised methods^26–28^ for HSI classification attracted strong interest. However, HSI with accurate annotation information are very rare in real life, it is necessary and very practical to study the unsupervised method for HSI classification. In the field of unsupervised HSI classification, in order to achieve accurate pixel-by-pixel classification, such as Bayesian estimation^29^ and sparse representation based techniques^30,31^ are applied. Because of the absence of label samples, most classifiers fail to achieve satisfactory classification performance due to the dimensionality problem. In addition, if the task is a pixel-level detection problem, as the question proposed in this research, existing HSI classification methods are more difficult to give an ideal performance.

Unlike the models mentioned above, in order to achieve the special purpose of this study, we focus on (1) spectral feature extraction and (2) density-based clustering in high-dimensional space. Firstly, for better spectral feature extraction and dimension reduction, we use PCA^32–35^ and SAE^36,37^ simultaneously to ensure the sparsity and representativeness. Then, in order to ensure the clustering result meet our flaking degree analysis task, we use the density-based OPTICS algorithm for clustering^38–40^. Since the number of specific categories is unknown before clustering, traditional clustering methods that need explicit number of classes show a bad performance when handling such uncertain detection task. Thus, we assume that the density of each pixel has a certain class in the aspect of the degree of flaking and use OPTICS to cluster. Finally, we calculate the distance in the feature space and visualize the final result using color map.

The contributions of our proposed method are highlighted as follows.

1. Studying the flaking degree of murals in Mogao Grottoes is very meaningful for the effective prevention of mural damage.
2. Considering the continuity of the degree of flaking, we use the density-based OPTICS algorithm for clustering. To the best of our knowledge, this is the first attempt to apply the algorithm to the field of HSI disease detection.
3. Both feature extraction and clustering are under unsupervised condition, which overcame the difficulty of not having any supervision information.
4. Visualizing the detect result of the flaking degree with the color map. Researchers can easily find areas of serious disease, which provides a useful suggestion for protecting the murals of Mogao Grottoes.

## Results

### Experimental data

**Figure 2:**
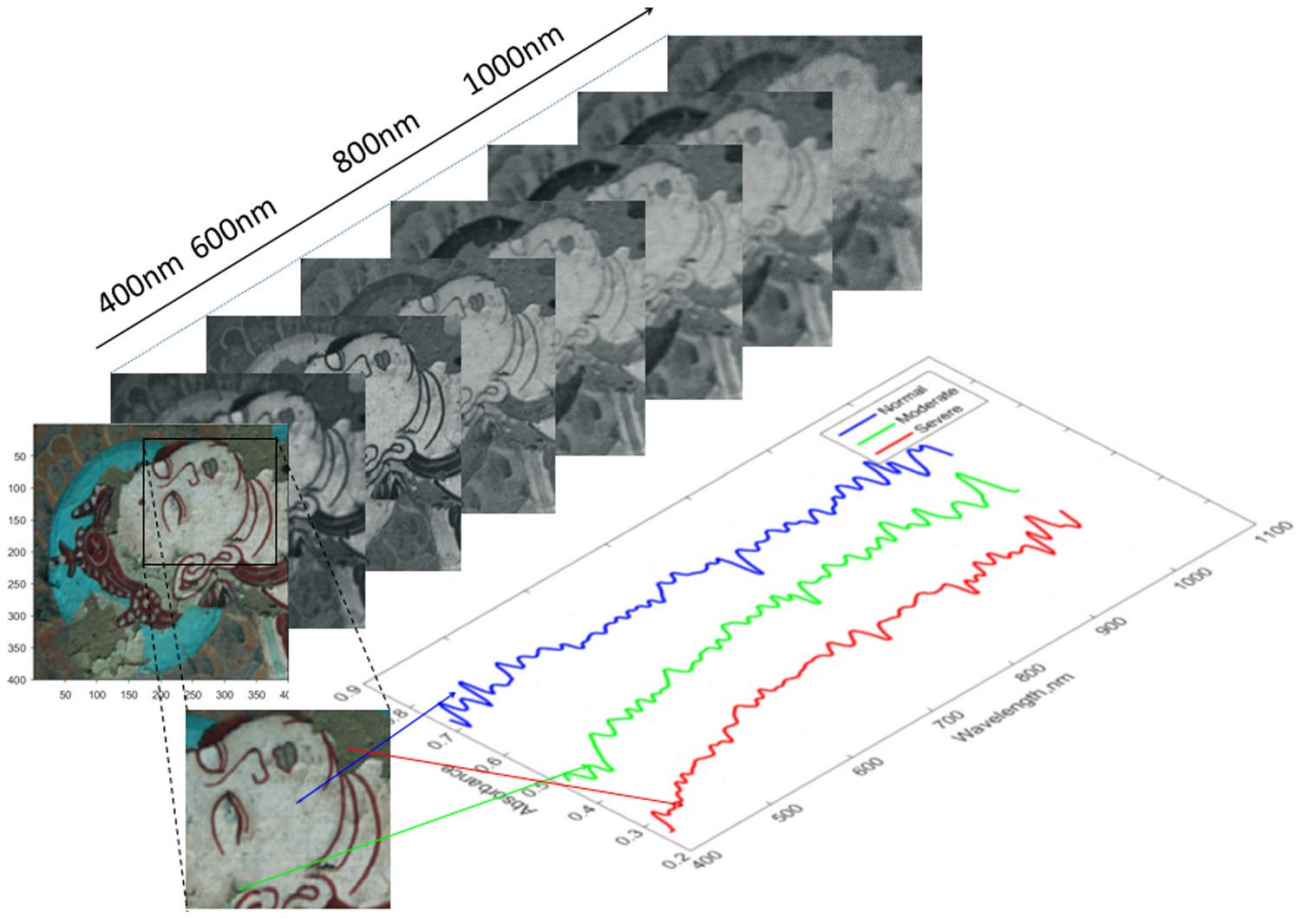
The HSI of the mural and corresponding spectral curves of different levels of flaking (three levels are defined in this image as normal, moderate and severe).

The HSI size obtained by V10-PS hyperspectral camera is 1000 × 960 × 728, where 728 is the number of spectral segments, and the whole HSI contains about one million pixels. Due to the huge amount of raw data, it will inevitably lead to time-consuming whether in feature extraction or in the clustering analysis process. So, in order to conveniently test the effectiveness of our method, we select two sub-graphs on the mural image as the test data, which space size are 250 × 250 and 400 × 400 respectively, and the spectral reflectance bands is 678 after discard the first 50 noise bands. Figure 2 shows the visualization images at different spectral bands of one sub-graph and displays the spectral information of three pixels representing three different degrees of flaking, represented by red, blue, and green, respectively.

### Comparison and analysis of the clustering result

**Figure 3:**
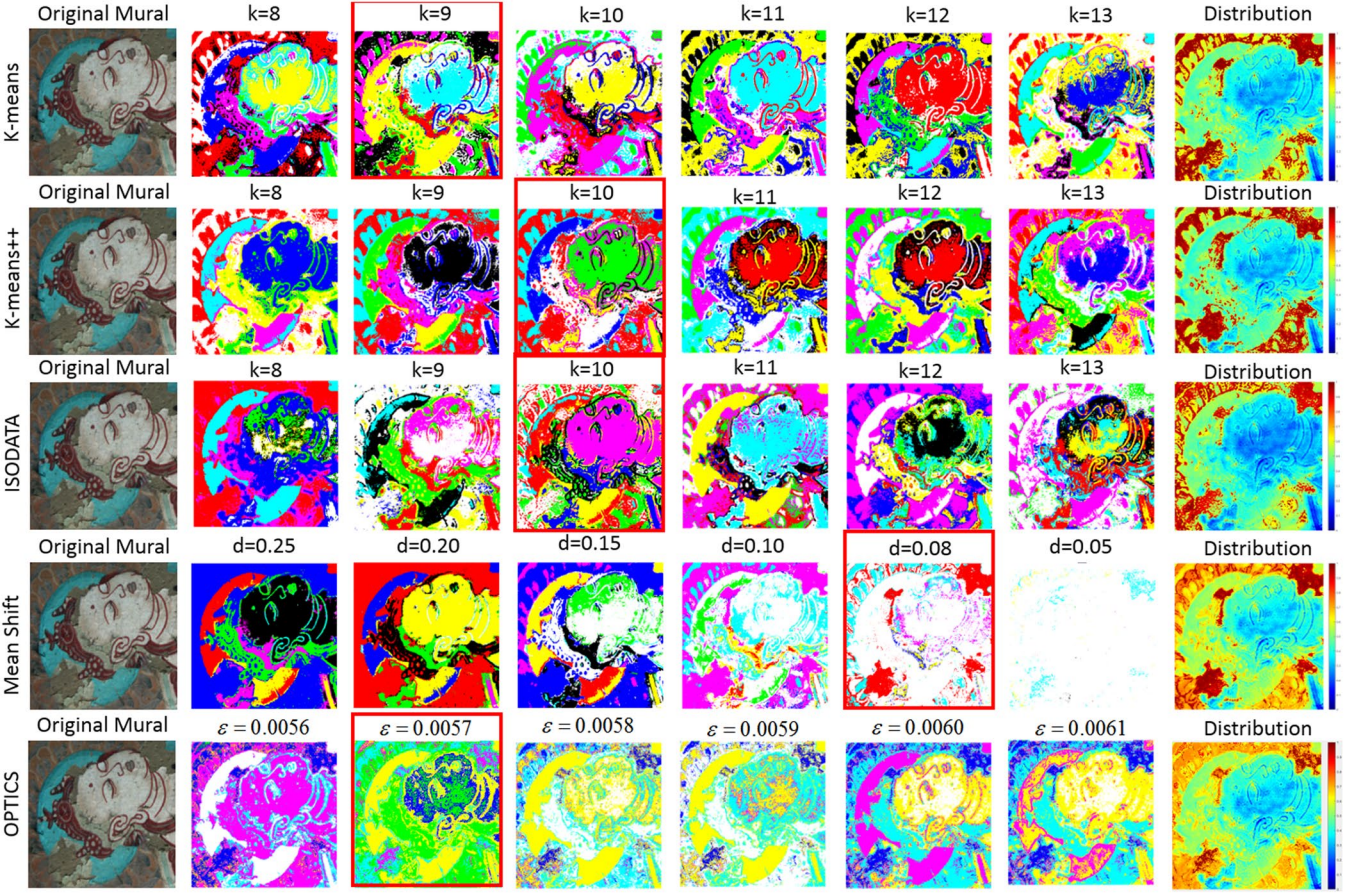
The clustering result of different methods under different parameter settings, and these clustering methods include K-means, K-means++, ISODATA, Mean Shift and OPTICS. The last column shows the final detection results for these five methods.

In order to prove the effectiveness and superiority of the clustering method based on OPTICS algorithm proposed in this paper when dealing with the mural data, we choose other four clustering algorithms to compare with OPTICS, including K-means^41^, K-means++ ^42^, ISODATA^43^ and Mean Shift (MS)^44^. In order to demonstrate the clustering result of different methods, we selected the data after SAE feature extraction as test data to be clustered. As shown in Fig. 3, the first four columns represent the clustering result of different methods under different parameter settings, and the last column shows the final detection results of flaking degree using different methods. Specifically, K-means, K-means++ and ISODATA need one input parameter, which is the final separable category number *k*. For MS, the input parameter is a threshold *d* from the center point. As for OPTICS, the two input parameters are a radius value *ɛ* and a minimum number of points *MinPts* in the circular area made up of the radius value *ɛ*, it is worth noting that OPTICS is not sensitive to these two parameters, they are only used as reference values. From the Fig. 3, we can draw conclusions: (1) K-means, K-means++ and ISODATA are not stable, their initial clustering centers are generated randomly, even if the input parameter *k* remains unchanged, the clustering results are different. (2) The calculation result of MS is stable. When the distance threshold *d* remains unchanged, the clustering result remains unchanged. But the fatal drawback of MS is that it is time consuming, especially when the number of pixels is large. (3) OPTICS is relatively stable. All the clustering results are obtained on the same output sequence. It only needs to take a certain amount of time to calculate the output sequence, and the clustering result remains the same if the input radius parameter is not changed. To sum up, compared with the other four methods, the OPTICS algorithm is not only stable, but also saves some time overhead.

In order to make more specific comparisons, we discuss as follows: we firstly choose a relatively good clustering result for each method, which is marked with a red box in Fig. 3. By observing the visualization of the clustering results and the final detection distribution map, we can find that both of these five methods can roughly complete the detection of flaking degree. But in detail, the detection results of the first four methods are not ideal, specifically, they detect the upper left and lower left corners of mural image as an area with serious flaking disease, but in fact, these two regions are relatively normal. The main reason lead to such results is that the spectral features of these two regions are very similar to those of the regions with severe flaking diseases, K-means, K-means++, ISODATA and MS failed to separate them clearly. Although the detection result of OPTICS is not completely correct, it can be seen through its clustering result where *ɛ* is 0.0057 that OPTICS can well separate the two regions from the complete flaking areas. The distance between them is amplified by OPTICS, which will reduce the influence of the pigment on the final detection result. In summary, OPTICS is more suitable as a clustering tool for our mural data.

### Performance and comparison

**Figure 4:**
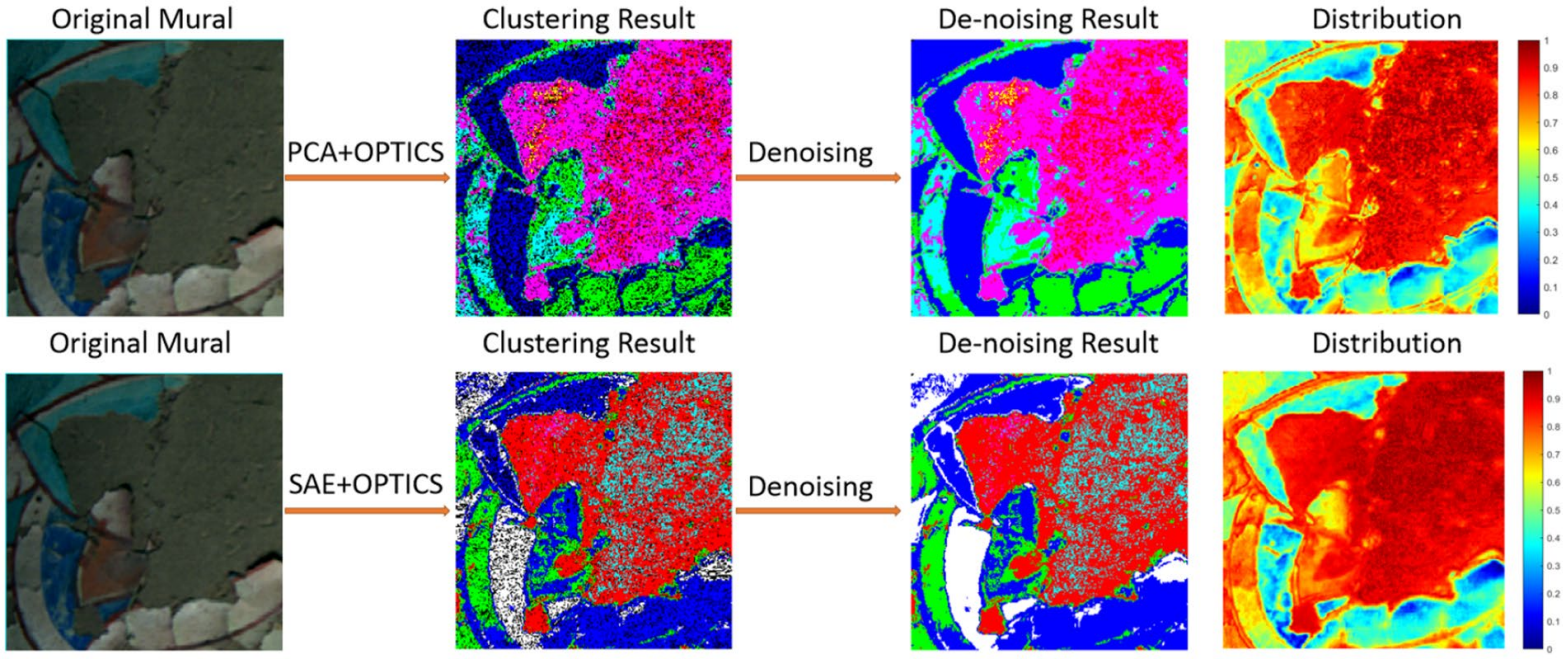
Experimental results of the area which has a large scale of mural flaking by using our proposed method.

**Figure 5:**
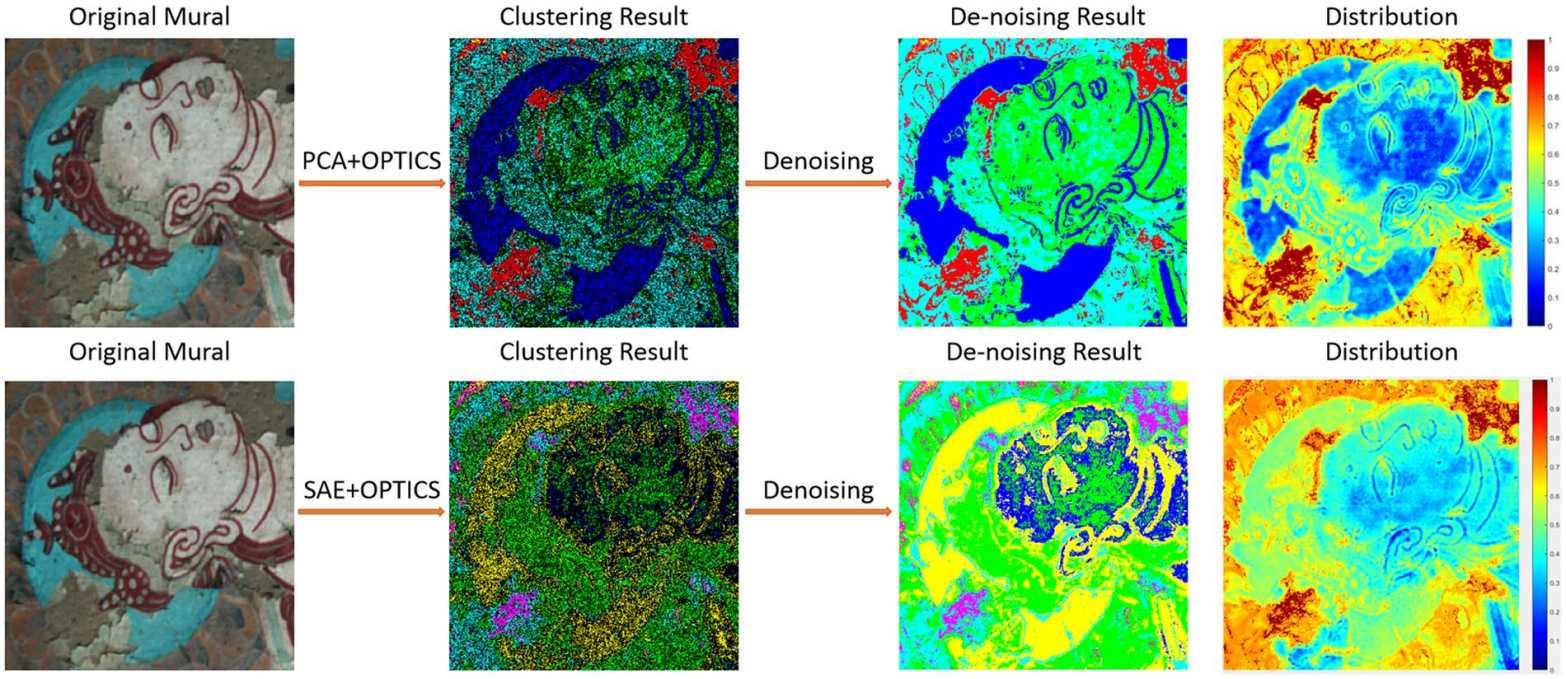
Experimental results of the area which has a small scale of mural flaking by using our proposed method.

The final detection results of the flaking disease severity are shown in Figs 4–5, where the sub-graph size used in Fig. 4 is 250 × 250 and in Fig. 5 is 400 × 400. As is shown both in Figs 4–5, the first row demonstrates the effect of PCA&OPTICS and the second row shows the effect of SAE&OPTICS. Specifically, the first column is the original mural image, the second column is the visualization of the clustering results and different colors represent different categories, in particular, the black dots demonstrate the noise. The third column shows the visualization result after eliminating noise points by using a denoising method, the idea of this method is to find the first non-noise point *j* in front of the noise point *i* in the cluster output sequence, then put the current noise point *i* into the category of the non-noise point *j*. The fourth column is the final detection result, specifically, the closer the region color is to red, the more severe is the flaking disease in the current region, whereas the closer the color is to blue, the lighter the degree of flaking.

Due to the complexity and continuity of the degree of flaking, it is difficult to obtain the correct distribution label through human marks, and in order to make objective and fair evaluation of the experimental results. We invited experts in the relevant fields to diagnose the extent of flaking of the murals and invited a large number of staff to evaluate our experimental results. By comparing the experts diagnosis results with our model detecting results, we can draw the following conclusions: (1) Both the PCA & OPTICS method and the SAE & OPTICS method show a good performance to highlight the areas which have severe flaking disease; (2) Compared with PCA, SAE is more effective and more representative for spectral feature extraction, and the SAE & OPTICS method has a better effect in detecting the degree of flaking of fine particles.

The visualization of the clustering results and the prediction of the distribution of flaking degree can provide useful information for researchers of Mogao Grottoes cultural relic protection, which shows the importance and significance of our work.

## Discussion

**Figure 6:**
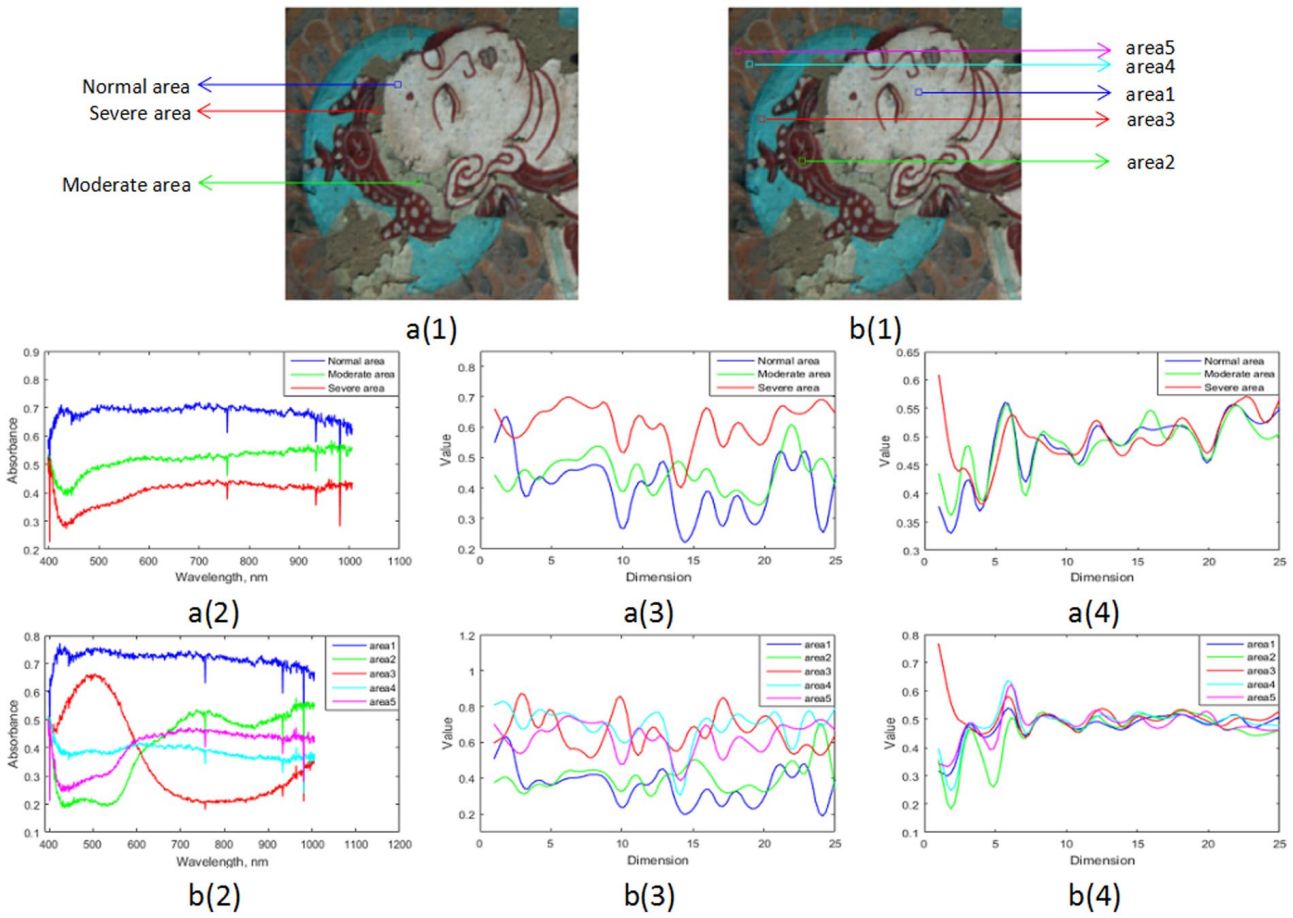
Average spectral curves for different areas. **a(1)** Shows the representative areas of the same pigment under different degree of flaking, and **b(1)** shows the typical area of different pigment in normal region. **a(2)** and **b(2)** Show the original spectral curves for the selected areas in **a(1)** and **b(1)**, respectively. **a(3)** and **b(3)** Show the spectral features after SAE extraction, **a(4)** and **b(4)** show the spectral features extracted by PCA.

Types of mural pigment are very rich, and different pigment corresponds to a different spectral curve. In Fig. 6, we compare the spectral features of the same pigment under different degrees of flaking, and the spectral features of different pigments in the normal region. a(1) shows the representative area of the same pigment under different degree of flaking, and b(1) shows the typical area of different pigment in normal region. In order to make the extracted spectral information sufficiently representative, we calculate the average spectral value of a local area with a size of 8 × 8 pixels.

As shown in Fig. 6, a(2) and b(2) show the original spectral curves for the selected areas in a(1) and b(1), respectively. From a(2), it can be seen that the areas with different degree of flaking have different spectral curves, and their distribution present a step shape, which provide some theoretical support for us to obtain the degree of flaking by calculating the distance between other point and the completely shedding center point in the feature space. b(2) shows that even if five blocks all belong to the normal area, different pigment offers absolutely different spectral feature, and this will inevitably have a certain degree of influence on the final detect results. a(3) and b(3) show the spectral features after SAE extraction. On the one hand, from a(3) we could see that after feature extraction and dimension reduction by SAE, the features still maintain the distribution shape of the original data, which shows that SAE does not lose important information of the original data, on the other hand, from the perspective of b(3), the spectral curves of different pigments in the normal area are closer, which shows that SAE will weaken the influence of pigments on the final detection results to a certain extent. For example, the spectral curves of area3, area4 and area5 are closer to each other after extraction, and the same phenomenon occurs in the spectral curves of area1 and area2. We calculate the average values of standard deviations for each dimension of data in b(2) and b(3), which are 0.1817 and 0.1914, and the difference is only 0.0103. Through the above analysis, we can find that SAE is effective enough to reduce the dimension, maintain the discrete degree of the whole data, and reduce the spectral information gap between different pigments as well.

a(4) and b(4) show the spectral features extracted by PCA. In order to display the trend of the curve, we select the data of the first 25 principal components. It can be seen from the curve that the data of the first three principal components help to distinguish the features of different area very well, as the curve extends backward, each feature curve starts to converge and shows the same trend, which is bad for distinguishing features of different regions. And the experiment shows that the representation coefficient of the first three principal components is more than 97% for the whole data, so we believe that the first three principal component data are sufficient to represent the overall data.

## Methods

### Mural data acquisition and calibration

**Figure 7:**
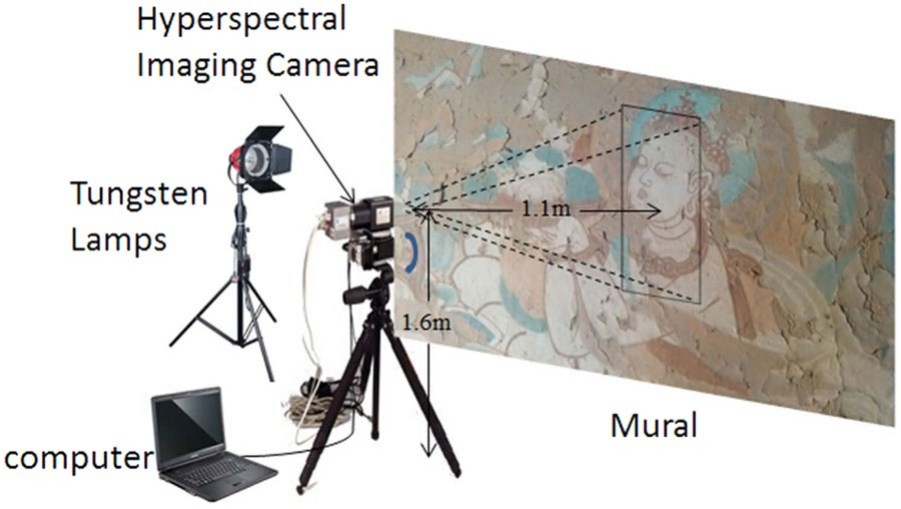
The hyperspectral imaging system.

The hyperspectral imaging system used in this study is shown in Fig. 7, the system contains hardware and software components. The hardware consists of a V10-PS hyperspectral camera, a mobile platform, a computer, and a tungsten filament searchlight which is used to provide light sources. The spectral range of V10-PS HSI camera is from 350 nm to 1006 nm with a spectral resolution 6.8 nm, and the spectral reflectance bands is 678 after discard the first 50 noise bands. All bands are mainly in the visible range with a few in the near infrared region. The size of the mural area covered is 1000 × 960 and the pixel size is 6.45 um × 6.45 um. The software system was to control the platform’s speed and the exposure time of the camera. The spectral camera was fixed to the mobile platform 1.6 m above the ground and 1.1 m from the mural.

We use the above-mentioned HSI camera to collect historic mural images of the Tang Dynasty in the 126th kiln of Mogao Grottoes. After finishing the collection of the mural, we gather the black current noise image (*B*) and white reference image (*W*) to correct the effects of the light sources. *B* is acquired by recording a spectral image when the light is off and camera lens is completely covered with a black cap, and *W* is obtained by acquiring a spectral image from a high reflectance white calibration tile. The HSI *T*_0_ was corrected by using a black reference and a white reference as follows:

1 $$T=\frac{{T}_{0}-B}{W-B}\times 100 \% $$

### OPTICS algorithm

Ordering Points to Identify the Clustering Structure (OPTICS) is one of the density-based cluster algorithm that was introduced by Ankerst, M.^38^, OPTICS is an extension of Density-Based Spatial Clustering Algorithm with Noise (DBSCAN) algorithm. The goal of OPTICS is to cluster the data according to the density distribution, owing to the output of the OPTICS is an orderly sequence, we can get the clustering result of any density from the sequence.

Given *P* as an object from database *DATA*;$\varepsilon$ is a distance value; *P* is called the core element, only if *P* satisfied the following formula (2), where *N*_*ε*_*(P)* is $\varepsilon$-neighbourhood of *P*; *MinPts* is the minimum number of points.

2 $${N}_{e}(p) > =MinPts$$

The core distance (CD) is determined for each core element of the dense region. CD is defined as the radius of the smallest area around a core element which contains *MinPts* points (including the core element itself)^45^. The CD is defined as following:

3 $$C{D}_{eMinPts}(p)=\{\frac{UNDEFINED,if\,{N}_{E}(p) < =MinPts}{MinPt{s}_{th}\,Dis\,\tan \,ce\,in\,{N}_{E}\,(p),else}\}$$

and the smallest reachable distance (RD) with respect to a core element is calculated for all points o of a dense region^45^. The RD of o with respect to *P* is defined as follows:

4 $$R{D}_{e,MinPts}(p,o)=\{\frac{UNDEFINED,if\,{N}_{e}\,(p) < MinPts}{\max (CD(p),dist(p,o)),else}\}$$

Through the OPTICS algorithm^46,47^, we can get the result queue, CD and RD of the original data. Then we can use these parameters and the radius to get the clustering result we need.

### The schematic of the proposed method

**Figure 8:**
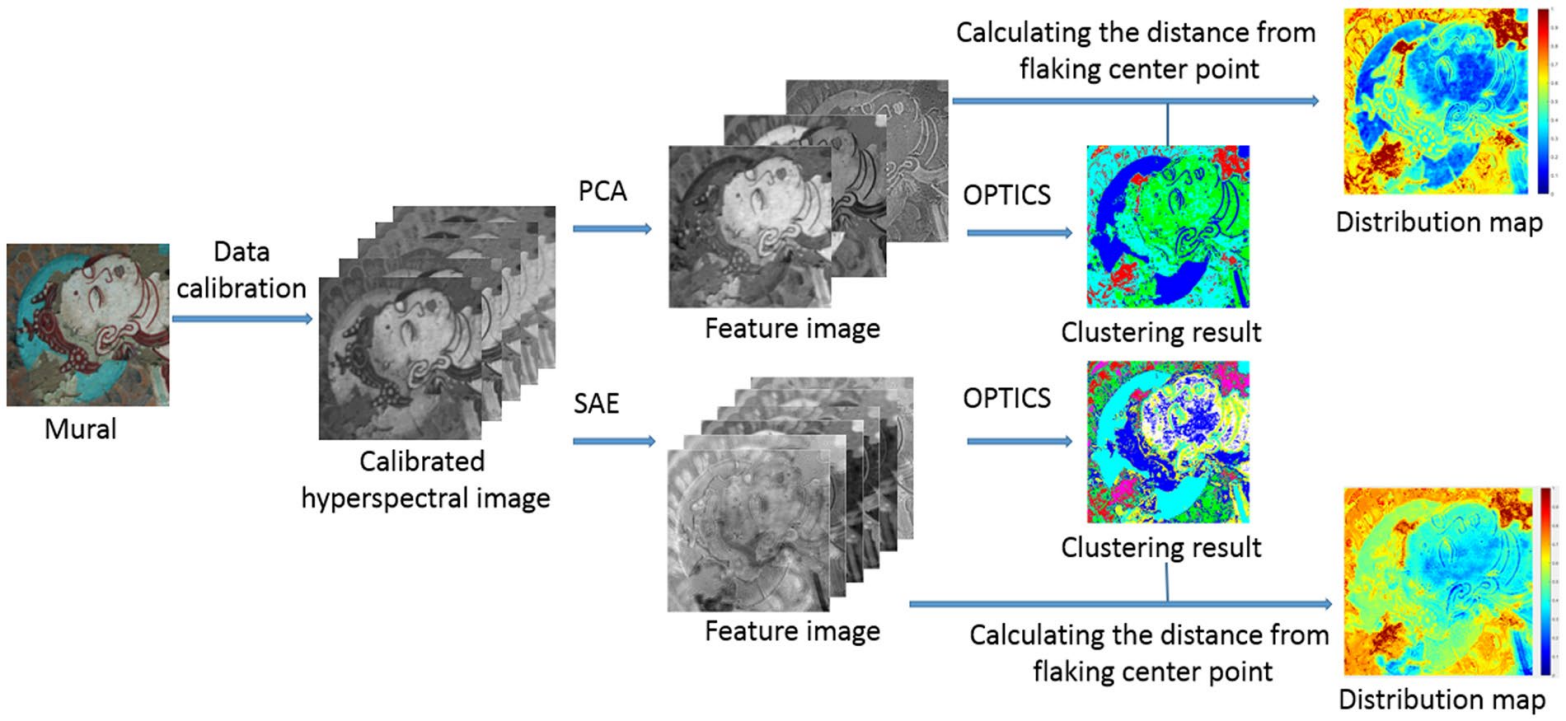
The schematic of the proposed method for evaluating the degree of flaking on murals at the Mogao Grottoes.

Figure 8 shows the schematic of the proposed method, which include the following steps: Firstly, we use V10-PS hyperspectral camera to capture the mural image and correct the effects of the light source. Secondly, the spectral features of the HSI are extracted by PCA and SAE respectively. Then, the extracted features are clustered by OPTICS. Finally, we calculate the Euclidean metric in feature space based on the result of OPTICS.

### Feature extraction

The mural HSI contain 728 spectral segments. To reduce the effect of noise, we exclude the first 50 bands. Nevertheless, the information contained in HSI is still large, which will inevitably lead to information redundancy and make the clustering operation too difficult to give an ideal result. Therefore, we use PCA and SAE to reduce the dimension.

#### PCA-based feature extraction

Because of its simple and parameter-less features, PCA is used as a standard tool for dimension reduction in a variety of HSI processing applications. What’s more, PCA is quite effective for extracting sparse and representative information of HSI. In this study, we use PCA for dimension reduction. We only use the first three dimensions of PCA results as spectral features because the information contained in the first three dimensions accounted for more than 97% of the original data after PCA operations.

#### SAE-based feature extraction

The pixels dimension D of the input data is 678, the number of hidden units M is 25, and the desired average activation λ of the hidden units is set to 0.01. After the operation of SAE, the dimension of the original data is downsized from 678 to 25. Therefore, SAE also plays an important role in dimension reduction.

### Feature clustering analysis

Before clustering, we normalized the features extracted by PCA and SAE respectively to ensure that the data has the same magnitude in different dimensions, which is beneficial to improve the experiments result. We set different radius to get different clustering result. Since the whole process is performed under unsupervised condition and the purpose is to detect the degree of disease under continuous change, it is hard to judge the clustering result very accurately. In addition, the mural pigments are diverse and complicated, it is hard to find the area which is completely free from flaking only by human eyes, so we finally choose the clustering result that can divide the macroscopic flaking area into the same category.

### Calculating the distance in feature space

First of all, we find the center point of the flaking area which is divided into the same class approximately, and define the center point as *cp*. Two different methods to find *cp* are as follows, method I aims to find a core point, which has the largest number of core points in the radius field; method II focus on constantly reduce the value of the radius, and take the final core point as the center point. Experimental results show that method II is more effective.

Secondly, if the point *p* and point *cp* are in the same class, we directly calculate the Euclidean distance between them and use it as the distance from the point of flaking disease. Otherwise, if *p* and *cp* are in different classes, then it means that those two points are not reachable in density, the direct calculation of the Euclidean distances between them is incomplete. We believe the distance between *p* and *cp* must be greater than a maximum, which is determined by *cp* and the points which belong to the same category as *cp*. The distance calculation method between points from different classes is concluded as follows:

5 $$Dis(p,cp)=\sqrt{\sum _{i=1}^{n}{({p}_{i}-c{p}_{i})}^{2}}+\,\max (\sqrt{\sum _{i=1}^{n}{({q}_{i}^{j}-c{p}_{i})}^{2}})(j=1,2,\ldots ,m)$$

Here *n* is the dimension of the pixel in the feature space, *q* is the collection of the points which are in the same class with *cp*, and *m* is the number of the collection and *q*^*j*^ means the *j*-th sample of the collection.
